# The Self Sabotaging Vessel: A Case Report and Literature Review of Spontaneous Coronary Artery Dissection

**DOI:** 10.7759/cureus.20835

**Published:** 2021-12-30

**Authors:** Esiemoghie J Akhigbe, Ebubechukwu Ezeh, Kanaan Mansoor, Jason Mader, Paul I Okhumale, Melissa Lester

**Affiliations:** 1 Internal Medicine, Marshall University Joan C. Edwards School of Medicine, Huntington, USA; 2 Cardiovascular Medicine, Marshall University Joan C. Edwards School of Medicine, Huntington, USA; 3 Cardiology, Marshall University, Huntington, USA; 4 Cardiology, Marshall University Joan C. Edwards School of Medicine, Huntington, USA

**Keywords:** spontaneous coronary artery dissection, primary percutaneous coronary intervention (pci), interventional cardiology, myocardial infarction, scad

## Abstract

Spontaneous coronary artery dissection (SCAD) is a very rare cause of acute coronary syndrome. Despite the recent advances in the management of cardiovascular diseases, the diagnoses and management of SCAD remain a dilemma. It has been described to majorly affect females of childbearing age, immediately post-partum or on oral contraceptives. Recent cases have also identified underlying connective tissue disease as a risk factor. Since its discovery, only a limited number of cases affecting males have been reported in the literature. This makes our case unique. In this, we present a 31-year-old male without any traditional risk factors who presented with atypical chest pain. Electrocardiogram showed ST-segment changes with echocardiogram revealing apical left ventricular akinesis. A diagnostic left heart catheterization showed multiple lumens in the distal left anterior descending artery (LAD). The patient was managed conservatively and discharged home on guideline-directed medical therapy.

## Introduction and background

Spontaneous coronary artery dissection (SCAD) is an important non-atherosclerotic, non-traumatic, non-iatrogenic etiology underlying acute coronary syndromes (ACS) or sudden cardiac death [1]. SCAD was first reported in 1931 [2]. Since then, there have been <1300 reported SCAD cases; most were published in the past five years [3]. In the general population, SCAD is the cause of ACS in 0.1 to 4 percent of cases [4]. Women with SCAD frequently present at a younger age (mean age of 38) and usually during the peripartum period or in association with oral contraceptive use with involvement of the left anterior descending (LAD) artery and/or left main coronary artery (LMCA), and SCAD usually occurs in the absence of traditional risk factors for coronary artery disease. In men, SCAD frequently presents at a slightly later age (mean age of 46), usually involves the right coronary artery (RCA), patients typically have clinical evidence of coronary artery disease (CAD) or risk factors for atherosclerosis [5]. This case illustrates an unusual presentation of SCAD in a young male with emphasis on the diagnosis, presentation, and management of this rare etiology of ACS.

## Review

Case presentation

The patient is a 31-year-old male with a past medical history of left popliteal artery occlusion status post mechanical thrombectomy, polysubstance abuse, nicotine addiction, chronic back pain secondary to trauma, anxiety, bipolar, and depression with multiple suicidal attempts. He presented on account of sudden onset of chest pain.

He described chest pain as sharp in nature, retrosternal in location, with each episode lasting for about 5 to 10 minutes. Chest pain was precipitated by emotional state with no known relieving factors. The pain was associated with diaphoresis and an episode of nausea and vomiting.

**Figure 1:**
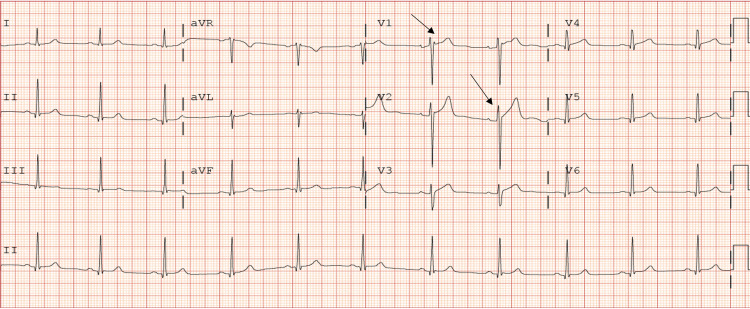
EKG showed sinus rhythm with black arrows indicating non-specific ST/T wave changes on V1-V2

**Figure 2:**
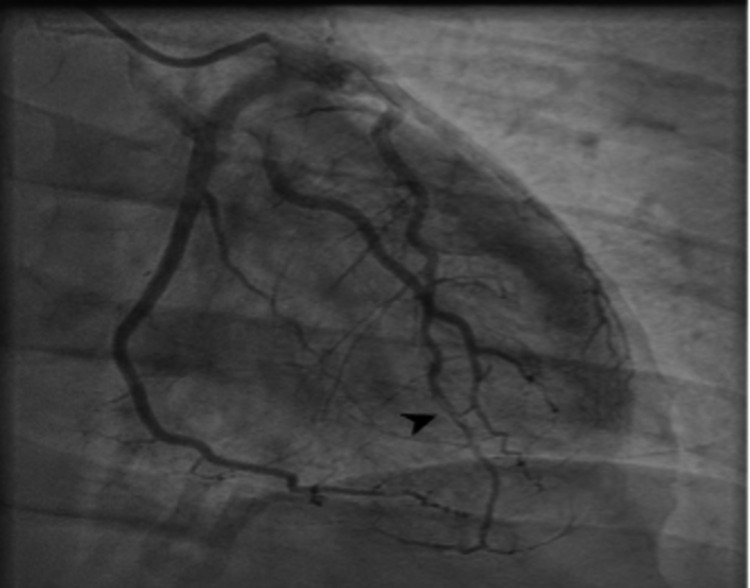
Cardiac catheterization showing a Type 1 spontaneous dissection of the distal left anterior descending artery

The physical exam was unremarkable. Initial laboratory findings include normal troponins at 0hr, 2hr, and 6hr. A urine drug screen that was positive for benzodiazepines, cannabinoids, and opiates. However, the patient explicitly denied any cocaine use and recent illicit drug use. Electrocardiogram performed showed sinus rhythm with non-specific ST/T wave changes minimal (Figure 1). An echocardiogram done on admission showed a normal-sized left ventricle, normal global systolic left ventricular function with a biplane EF of 51%, apical septal and apical left ventricular wall segment were akinetic. A diagnostic cardiac catheterization was performed to define the anatomy and showed multiple luminal defects in the distal left anterior descending artery (Figure 2) with no other significant obstruction. A presumptive diagnosis of Type 1 spontaneous coronary artery dissection was made. The patient was managed conservatively and started on ASA, Beta-blockers. The patient’s symptoms improved markedly. He was eventually discharged to a cardiac rehabilitation program with a close outpatient follow-up.

Review

The definition of SCAD has evolved over the years, with the first case report by Pretty in 1931 describing a dissected coronary artery atherosclerotic aneurysm [4]. Spontaneous coronary artery dissection (SCAD) has been described as an uncommon and heterogeneous condition, most commonly presenting with acute coronary syndrome (ACS) [6]. After it was first described, about 150 cases of SCAD were reported in the next 50 years in the scientific literature [2].

SCAD is defined as a nontraumatic, non-iatrogenic, and nonatherosclerotic separation of the coronary arterial wall by an intramural hematoma (IMH), creating a false lumen, which then compresses the true lumen, causing myocardial ischemia or infarction. The separation can occur between the intima, media, or adventitia and can originate from an intimal tear leading to dissection into the arterial wall or may result from spontaneous bleeding from ruptured vasa vasorum without intimal tear [7,8].

The true prevalence of the condition is unknown because of underdiagnosis, but it has been reported in 0.1% to 4.0% of patients presenting with acute coronary syndrome. In a large National Inpatient Sample analysis from 2009 to 2014, among about 750,000 women who presented with myocardial infarction (MI) and underwent coronary angiography, about 1% were reported to be due to SCAD [3]. Cohort studies incorporating direct angiographic review indicate that one quarter to one-third of myocardial infarctions in women younger than 50 years of age are caused by SCAD [9]. In a study by Krittanawong et al., of a total of 30,425 ACS patients, 375 (1.2%) SCAD patients presented with ACS and the mean age of the SCAD patients was 52.2 ± 12.8 years, 64.3% were female and 44% were white. Of total, 11.4% of SCAD patients died and 8.8% of non-SCAD ACS patients died [1].

The exact pathophysiology of SCAD remains unclear and has been postulated to involve multiple predisposing or precipitating factors including genetic abnormalities, inherited or acquired vasculopathies, hormonal influences, inflammation, intense exercise, emotional stress, and recreational drugs [10].

In recent literature, two principal mechanisms for the etiology of SCAD have been proposed. Firstly, the “inside-out” mechanism proposes that a “tear” in the endothelium-intima layer allows blood to accumulate in the media; whereas the “outside-in” mechanism initiates the event, perhaps consisting of microvessel rupture that occurs within the vessel wall [11,12].

The etiology of SCAD is multifactorial. Numerous case reports have described an association between SCAD and several inflammatory disorders such as systemic lupus erythematosus, sarcoidosis, inflammatory bowel disease, and celiac disease; however, registry data indicate that the prevalence of systemic inflammatory disorders among patients with SCAD is low (<5% in most cohorts) [13]. SCAD has also been indicated to be associated with emotional stress, the presence of fibromuscular dysplasia (FMD), chronic kidney disease (CKD), chronic obstructive pulmonary disease (COPD), peripheral artery disease (PAD), and carotid artery disease [1].

Although SCAD can affect any coronary artery or its branches, the most common artery affected is the LAD, which supplies blood to the front (anterior) part of the heart. This is like the patient described in our case who on coronary angiography had SCAD of the LAD. Other coronary artery branches may also be affected by SCAD, and more than one vessel may be affected by SCAD at the same time. The location of the arteries affected by SCAD and the severity of the blockage(s) determine the severity of symptoms [2,14,15].

The age of presentation is variable in males and females with women frequently presenting at a younger age (mean age of 38) and usually during the peripartum period or in association with oral contraceptive use with involvement of the left anterior descending (LAD) artery and/or LMCA (left main coronary artery) are most involved. Men, however, present at a slightly later age (mean age of 46), usually with involvement of the right coronary artery (RCA), with evidence of CAD or risk factors for atherosclerosis [5].

As with other causes of ACS, SCAD presents classically with chest pain, shoulder, or epigastric pain (96% of cases), with or without radiation to the arm (52%), less frequently it presents with nausea or vomiting (24%), radiation to the neck (22%), diaphoresis (21%), dyspnea (20%) and infrequently with back pain, dizziness, fatigue, headache or syncope (<20% each) [16]. Importantly, even cardiogenic shock or sudden cardiac death could be the initial presentation of SCAD [17]. Laboratory changes in SCAD mimic that of ACS, it is usually associated with a modest elevation of cardiac enzymes. EKG changes such as ST-segment elevation and non-ST-segment elevation myocardial infarction vary in frequency between different series, but the full spectrum of ischemic disease is represented in SCAD patients.

**Figure 3:**
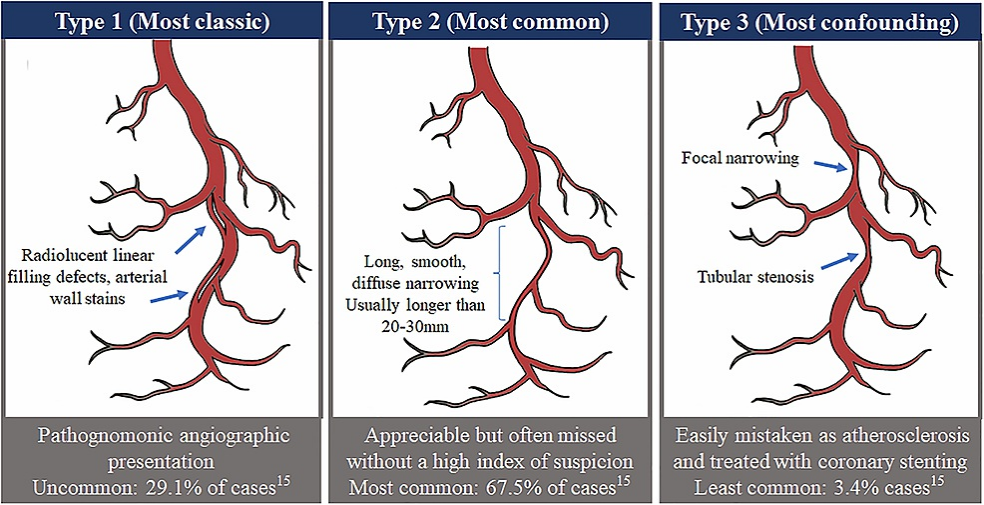
Diagram showing the angiographic classification of spontaneous coronary artery dissection (SCAD) Reproduced with permission from Low et al.

Accurate and early diagnosis of SCAD is important because the management and investigation of SCAD are different from atherosclerotic disease. Coronary angiography is widely available and is the first-line imaging for patients presenting with ACS [18]. Historically the gold standard for the diagnosis of SCAD has been by coronary angiography. SCAD has been described by three (3) distinct angiographic patterns (Table 1); Type 1 (evident arterial wall stain), typical contrast dye staining of the arterial wall is depicted along with visible multiple radiolucent lumens. Type 1 SCAD appearance is considered pathognomonic and corresponds to approximately 25% of all cases. Type 2 represents about 70% of all patients angiographically diagnosed with SCAD. Type 2 angiographic SCAD describes a long diffuse (typically >20 mm) stenosis that varies in severity from mild to complete occlusion. This appearance commonly involves the mid-segment and often extends to the distal segment and tip of the arteries with dissection and/or wall hematoma formation producing smooth coronary artery narrowing. Type 2 SCAD is further classified in type 2A (normal arterial segment distal to the dissection) and type 2B (dissection extends to the distal tip of the coronary artery). Often unnoticed or misdiagnosed, type 2 SCAD can cause moderate stenosis and as much as complete occlusion. Finally, type 3 SCAD mimics atherosclerosis and is the most challenging to recognize. Its differentiation from atherosclerotic stenosis is very difficult and usually requires intracoronary imaging (Figure 3) [7,18].

**Table 1:** Angiographic classification of spontaneous coronary artery dissection (SCAD) Classification of SCAD according to Saw et al. [7]

TYPE OF SCAD	PERCENTAGE OF CASES	ANGIOGRAPHIC DESCRIPTION
Type 1	29-48%	False Lumen with filling defects
Type 2A	50-70%	Diffuse stenosis of varying length with intramural hematoma (IMH) narrowing being bordered by normal artery segments both proximally and distally
Type 2B	Same as above	IMH narrowing located at the apical tip of the artery
Type 3	2-4%	Focal and tubular stenosis (length typically <20mm) that mimics atherosclerosis

Despite its widespread use in the diagnosis of SCAD, coronary angiography has significant limitations. This is because coronary angiography is a two-dimensional luminogram that does not image the arterial wall. This makes the diagnosis of SCAD by angiography challenging to almost impossible in the absence of pathognomonic multiple radiolucent lumens with contrast staining of the arterial wall. For this reason, intracoronary imaging with optical coherence tomography (OCT) or intravascular ultrasound (IVUS) is the true gold standard for SCAD diagnosis [7,18,19].

Optical coherence tomography is a more advanced technology that uses light waves to image the arterial wall. This technology has revolutionized the diagnosis of SCAD since its first reported use in 2008 [9]. It has a spatial resolution of 10 to 20 μm, and thus, it is superior to intravascular ultrasonography for delineating the lumen-intimal interface and is better for visualizing intimal tears, false lumen, IMH, and intraluminal thrombi. This superior imaging improves the ease of detecting SCAD and is the preferred intracoronary imaging method when an angiographic diagnosis is uncertain [18]. Cardiac computed tomography (CT) angiography (CCTA) has much lower spatial resolution compared with conventional angiography and does not allow visualization of flow. It is not recommended as a first-line diagnostic test for suspected SCAD and is contraindicated in high-risk ACS cases [12].

The treatment of SCAD is controversial but current recommendations on management are largely used based on expert opinions from observational series. Medical therapy such as Beta-blockers, antiplatelet therapy, anticoagulation, and cholesterol-reducing agents have been studied. Beta-blockers play an important role in the pharmacological arsenal for SCAD. Its role in the reduction of coronary arterial wall stress and in reducing ventricular arrhythmias has been proven to provide long-term survival benefits [20].

The role of antiplatelet therapy for SCAD is unknown, but based on recent literature, the use of Aspirin (ASA) in ACS and secondary prevention, together with its low side effect profile, makes it a reasonable use for acute and long-term SCAD management. Clopidogrel for acute management of SCAD patients not treated with stents is of uncertain benefit. However, considering that a proportion of SCAD involves intimal tears, which can be prothrombotic, dual antiplatelet therapy (DAPT) could be empirically beneficial [15,20].

The use of anticoagulant and thrombolytic therapy for SCAD is controversial and has not been well studied. The clinical benefit of heparin in SCAD has not been established but it is routinely administered for ACS management in hospitals. There is a potential risk of extending the dissection with anticoagulation, which is balanced by the potential benefit of resolving overlying thrombus and improving true lumen patency [3]. In a retrospective review of 87 SCAD patients who received thrombolysis, 60% had clinical deterioration that required rescue percutaneous coronary intervention (PCI) or coronary artery bypass grafting (CABG). Thrombolytic therapy should be avoided in SCAD because there have been reports of harm and clinical deterioration due to the extension of IMH and dissection [20].

The use of statins for SCAD is controversial. A small retrospective study demonstrated potentially higher SCAD recurrence with statins; however, the bulk of data for ACS demonstrates significant benefit with lipid-lowering, and statins are routinely recommended post-MI [17]. However, because of the lack of atherosclerosis in SCAD patients, statins tend to only be administered to patients with pre-existing dyslipidemia [17,21].

Most cases of SCAD patients who are hemodynamically stable are managed conservatively. This approach relies on observations that SCAD arteries heal spontaneously in most cases, and that revascularization is associated with high failure rates [4,21,22].

In a retrospectively reviewed study, a cohort of patients with nonatherosclerotic SCAD were prospectively followed at Vancouver General Hospital and underwent repeat coronary angiography following the index SCAD event. Of the 182 patients who underwent repeat coronary angiography after the index SCAD event, about 157 (86.3%) of them had ﻿spontaneous angiographic healing. Spontaneous healing was described as meeting the following three criteria: improvement of stenosis severity from index event, residual stenosis <50%, and TIMI (thrombolysis in myocardial infarction) flow grade 3 [4].

When spontaneous rupture affects major arteries such as the left main or proximal segments of the LAD, PCI should be attempted. In selected patients, PCI or coronary artery bypass grafting (CABG) is considered; indications for revascularization include left main coronary artery or severe proximal 2-vessel dissection, ongoing ischemic symptoms, hemodynamic instability, cardiogenic shock or ventricular arrhythmias [18]. In recent studies, only about half of the revascularization attempts via PCI were successful in SCAD, while the risk of iatrogenically induced worsening of the dissection was very high [18,19,23]. Recurrent SCAD is well recognized. Report incidences range from 5% to 19% of cases [24]. After the acute phase, estimated survival is good, at about 70% to 90% [25].

## Conclusions

SCAD is a rare but potentially deadly cause of acute coronary syndrome. This condition has been extensively described to affect mainly females thus making a diagnosis in males an enigma. Fewer than 200 cases of SCAD have been reported in the literature, and the majority (80%) have occurred in young women. This unusual presentation of SCAD in this patient could be a cause of numerous missed diagnoses, leading to an increased mortality and morbidity in this demographic. In view of the high rates of major adverse cardiac events in the long term, close follow-up is recommended for patients who suffer a SCAD. It is also important for physicians to have a high index of suspicion when presented with patients without traditional risk factors for CAD presenting with chest pain.
